# Improved Thermochemical Energy Storage Behavior of Manganese Oxide by Molybdenum Doping

**DOI:** 10.3390/molecules26030583

**Published:** 2021-01-22

**Authors:** Javier Moya, Javier Marugán, María Orfila, Manuel Antonio Díaz-Pérez, Juan Carlos Serrano-Ruiz

**Affiliations:** 1 Department of Chemical and Environmental Technology, Universidad Rey Juan Carlos, C/Tulipán s/n, 28933 Móstoles, Madrid, Spain; j.moyas@alumnos.urjc.es (J.M.); javier.marugan@urjc.es (J.M.); maria.orfila@urjc.es (M.O.); 2 Materials and Sustainability Group, Department of Engineering, Universidad Loyola Andalucía, Avda. De las Universidades s/n, 41704 Dos Hermanas, Seville, Spain; madiaz@uloyola.es

**Keywords:** thermochemical energy storage, Mn–Mo mixed oxides, reducibility, redox cyclability, oxidation kinetics

## Abstract

To improve the thermochemical energy storage (TCS) behavior of Mn_2_O_3_, several Mn–Mo oxides with varying amounts of MoO_3_ (0–30 wt%) were prepared by a precipitation method. The physico-chemical properties of the solids were studied by N_2_ adsorption–desorption, X-ray diffraction (XRD), scanning electron microscopy (SEM), and H_2_-temperature-programmed reduction (TPR), while their TCS behavior was determined by thermogravimetric analysis coupled with differential scanning calorimetry (TGA-DSC). Apart from Mn_2_O_3_ and MoO_3_ phases, XRD revealed a mixed MnMoO_4_ phase for MoO_3_ loadings equal or higher than 1.5 wt%. All samples showed a well-formed coral-like surface morphology, particularly those solids with low MoO_3_ contents. This coral morphology was progressively decorated with compact and Mo-enriched MnMoO_4_ particles as the MoO_3_ content increased. TPR revealed that the redox behavior of Mn_2_O_3_ was significantly altered upon addition of Mo. The TCS behavior of Mn_2_O_3_ (mostly oxidation kinetics and redox cyclability) was enhanced by addition of low amounts of Mo (0.6 and 1.5% MoO_3_) without significantly increasing the reduction temperature of the solids. The coral morphology (which facilitated oxygen diffusion) and a smoother transition from the reduced to oxidized phase were suggested to be responsible for this improved TCS behavior. The samples containing 0.6 and 1.5 wt% of MoO_3_ showed outstanding cyclability after 45 consecutive reduction–oxidation cycles at high temperatures (600–1000 °C). These materials could potentially reach absorption efficiencies higher than 90% at concentration capacity values typical of concentrated solar power plants.

## 1. Introduction

Global energy consumption is increasing by the ever-growing demand of energy from developing countries, and renewable energy sources are becoming more important in covering this demand [1]. Among the renewable energy technologies available today, concentrated solar power (CSP), with an installed generation capacity of 4.4 GW in 2014 [2], is particularly relevant. As is common to renewable energies, the discontinuous nature of solar energy makes necessary the development of storage technologies to overcome the negative effects of transitory weather conditions, to modulate low-to-high energy gaps, and to achieve continuous 24 h electricity production [3,4]. In the case of CSP, the development of efficient thermal energy storage (TES) systems is particularly important. Three different TES approaches based on sensible, latent, and thermochemical heat are currently available. Among them, thermochemical energy storage (TCS, i.e., the utilization of a reversible chemical process to store thermal energy) holds great promise for future CSP plants owing to its higher energy density and discharge temperatures compared to sensible- and latent heat-based approaches. While high discharge temperatures allow increasing the efficiency of steam turbines, high energy densities are important for reducing the size of the storage tank facilities and, therefore, decreasing the capital expenses of the plant [3,5,6]. TCS allows solar thermal energy to be stored (and subsequently released) in form of chemical energy by promoting a high-temperature reversible chemical process. Thus, a highly endothermic reaction is used for the charging step, while the reverse exothermic process allows releasing energy in the discharge step while closing the chemical loop for a new cycle. As chemical bonds are broken and formed during the process, large amounts of energy are potentially absorbed/released within each cycle [7,8].

A good number of solids including hydrides, hydroxides, carbonates, and oxides have been tested as TCS materials. Among them, metal oxides are particularly interesting because of their high reaction temperatures and the utilization of oxygen as a reactant, which opens the possibility of using air as a working fluid and thus simplify gas storage installations [5,6,9]. General Atomics conducted a comprehensive analysis on the main TCS technologies with potential to be used in CSP plants, covering a large number of metal oxides [10]. This study identified cobalt (II,III) oxide (Co_3_O_4_) as the most promising TCS material in virtue of its superior energy density, high conversion temperature, and good cyclability. Despite its demonstrated thermal stability [11], Co_3_O_4_ is costly and toxic and, therefore, less harmful and cheaper alternatives are required. In this sense, other oxides such as CuO have shown superior energy density and reaction temperatures [11]. However, the reduction temperature of CuO is close to the melting point of its reduced form (Cu_2_O), which increases the risk of sintering and loss of activity [11,12,13]. Barium oxide (BaO), despite having a reaction temperature well suited for superheated steam cycles, suffers from poor thermal stability and high sensitivity to water and CO_2_ traces (leading to inactive hydroxide or carbonate materials, respectively) [6,11,14,15,16].

Manganese (III) oxide (Mn_2_O_3_) has been widely studied as a TCS material [17] in virtue of the following charge and discharge processes:

Charge (reduction): 6 Mn_2_O_3_ → 4 Mn_3_O_4_ + O_2_ (1)

Discharge (oxidation): 4 Mn_3_O_4_ + O_2_ → 6 Mn_2_O_3_ (2)

Despite its low cost and abundancy, Mn_2_O_3_ suffers from a number of drawbacks. Thus, the reduced form of Mn_2_O_3_ (Mn_3_O_4_) has been reported to show very low re-oxidation rates, which leads to incomplete regeneration of the original oxide and poor cycling behavior [10,17]. These limitations can be overcome by enhancing the redox behavior of Mn via formation of a mixed oxide [13,18,19,20]. Among the large number of combinations tested, Mn–Fe mixed oxides have shown good oxidation behaviors and faster re-oxidation rates compared to Mn_2_O_3_. However, these improvements are achieved at the expense of increasing the reduction (charge) temperature of the resulting mixed oxides, which complicates the CSP process [8,10,13,21,22]. To overcome this issue, we suggest herein, for the first time, the utilization of Mo in replacement of Fe to improve the TCS properties of Mn_2_O_3_ without significantly increasing the reduction temperature of the solids. Mo was selected because it possesses very similar size (ionic radius of Mo^6+^: 73 pm) compared to Mn^3+^ (72 pm), thereby facilitating the substitution process. In fact, Mn and Mo have been previously described to form stable mixed-oxide phases such as MoMnO_4_ and Mo_3_Mn_2_O_8_ [23,24], and Mo^6+^ has been reported to substitute Mn^3+^ by forming six-fold octahedral coordination compounds [25,26,27]. Having this in mind, we herein analyzed the suitability of Mo-doped Mn_2_O_3_ as TCS materials. Several Mo-doped manganese oxides with varying loadings of Mo were prepared by a precipitation method. The textural, structural, morphological, redox, and thermochemical properties of these materials were characterized by N_2_ adsorption–desorption, X-ray diffraction (XRD), scanning electron microscopy (SEM), H_2_-temperature-programmed reduction (TPR), and thermogravimetric analysis coupled with differential scanning calorimetry (TGA-DSC), respectively.

## 2. Materials and Methods

### 2.1. Materials Preparation

Mn–Mo solids with varying MoO_3_ loadings (0, 0.6, 1.5, 8.7, 12, and 31 wt%, denoted as X% MoO_3_ with x indicating the MoO_3_ loading) were prepared by a precipitation method. Briefly, appropriate amounts of the precursors Mn(NO_3_)_2_·4H_2_O (Scharlau, Barcelona, Spain, >98.5%) and (NH_4_)_6_Mo_7_O_24_·4H_2_O (Sigma-Aldrich, St. Louis, MO, USA, >99.0%) were dissolved in 250 mL of distilled water and stirred for 30 min. A 2.5 M NaOH aqueous solution was subsequently added dropwise until pH = 12 was reached. This pH was maintained for 4 h while stirring, and the as-obtained precipitate was subsequently filtered and cleaned three times with 500 mL of Milli-Q^®^ water. The as-obtained solid was subsequently dried at 100 °C overnight, ground to fine powders with an agate mortar, calcined at 750 °C under static air for 4 h (10 °C/min heating rate), ground again, and finally stored for analysis.

In addition, the 100% MoO_3_ sample was prepared by directly calcining (NH_4_)_6_Mo_7_O_24_·4H_2_O at 350 °C for 4 h. The calcined sample was ground in an agate mortar. This material was not characterized in full since it sublimates at 600 °C [28].

### 2.2. Materials Characterization

TPR experiments (ca. 45 mg of sample) were performed on a Micromeritics Autochem II within a temperature range of 60–900 °C (10 °C/min heating rate) under flowing (50 mL/min) 10%H_2_/Ar. The specific surface areas of the samples were obtained by adsorption–desorption of N_2_ at 77 K on a Micromeritics ASAP 2020 following the multi-point Brunauer–Emmett–Teller (BET) method. The samples were previously degassed at 300 °C under vacuum for 100 min. Powder XRD analyses were carried out on a Rigaku UltimaIV diffractometer employing CuKα radiation and a scanning rate of 2°/min. SEM observations were carried out on a Hitachi S5200 microscope, and samples were used without any further modification. Inductively coupled plasma atomic emission spectroscopy (ICP–AES) was used to determine the actual oxide content of the different samples. This measurement was made on a Varian Vista AX spectrometer, and samples were previously dissolved in 5 mL of aqua regia (HNO_3_:HCl, 1:3 in volume).

### 2.3. Thermal Cyclability

The TCS behavior of the samples upon repetitive thermal cycles was studied by TGA-DSC on a TA Instruments SDT Q-600 device. The samples (18–22 mg) were placed on 90 μL alumina crucibles. At least 10 redox cycles were performed over each sample under flowing air (50 mL/min, oxidation) at temperatures ranging from 600 to 1000 °C.

## 3. Results and Discussion

### 3.1. Thermodynamic Study

We considered the following process to describe the thermochemical behavior of Mn–Mo mixed oxides:

(3) $${\mathrm{Mo}}_{x}{\mathrm{Mn}}_{2-2\cdot x}O_{3}\to {\mathrm{Mo}}_{x}{\mathrm{Mn}}_{2-2\cdot x}O_{2.67-0.67\cdot x}+\frac{0.33+0.67\cdot x}{2}O_{2}$$

**Figure 1:**
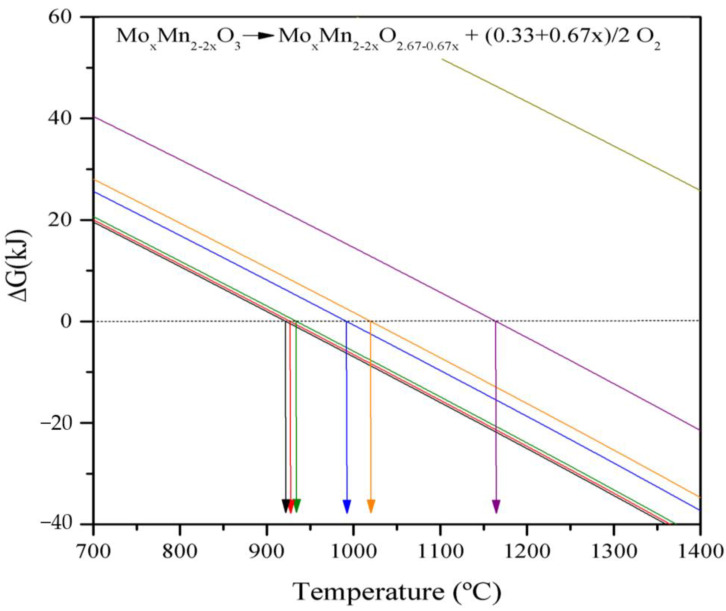
Theoretical evaluation of the reduction temperature of the samples. From bottom to top 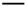:0%MoO_3_; 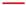: 0.6%MoO_3_; 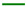: 1.5%MoO_3_; 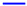: 8.7%MoO_3_; 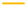: 12%MoO_3_; 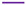: 31%MoO_3_; 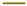: 100%MoO_3_.

The process was theoretically studied using the HSC Chemistry 6.1 software @Outotec Research Oy. The dependence of the Gibbs free energy of reaction (∆G_r_^T^) with the temperature was studied. This energy was calculated from the standard enthalpies (∆H^°^_f_) and entropies (∆S^°^_f_) of formation for the pure oxides (Mn_2_O_3_ and MoO_3_) and the temperature dependence of its specific heat (Cp(T)). The data for the mixed oxide samples were obtained by interpolating pure oxides at their corresponding content (Figure 1), and the results are summarized in Table 1. As shown in Table 1 and Figure 1, the reduction temperature increased significantly with the amount of Mo in the mixed oxide. This theoretical evaluation assumed the formation of a mixed oxide phase having intermediate physical–chemical properties mixed as compared to the pure manganese or molybdenum oxides.

### 3.2. Physical–Chemical Characterization

The morphology, composition, and crystal structure of the samples were characterized by a number of techniques. As shown in Table 1, the actual Mo contents of the samples (determined by ICP-AES) were very similar to the nominal values. All the samples showed low BET surface areas and low porosity, probably as a result of the high calcination temperature used herein (750 °C). These samples are expected to undergo sintering during thermal cycling, decreasing the surface area below the detection limit of the adsorption device.

**Figure 2:**
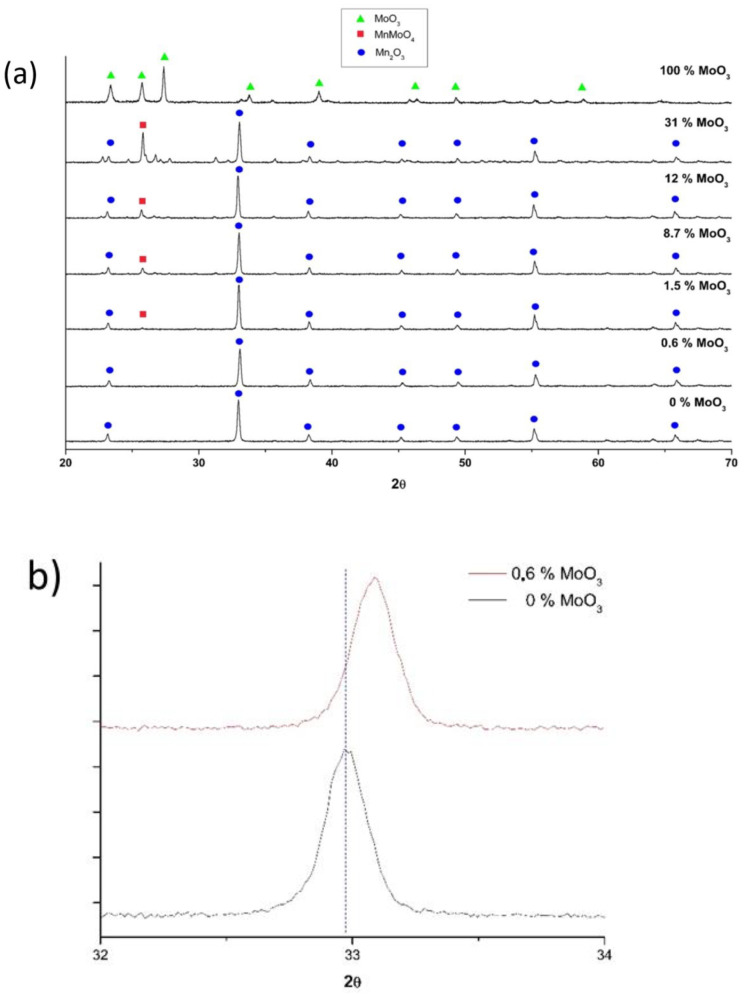
(**a**) XRD patterns for the X%-MoO_3_ samples. Three phases were identified: Mn_2_O_3_, MnMoO_4_, and MoO_3_. (**b**) Zoomed 2θ region 32–34° for the samples 0% MnO_3_ and 0.6% MnO_3_.

Figure 2a shows the XRD patterns of all the oxides under study. The diffractograms revealed the presence of a cubic Mn_2_O_3_ crystalline phase (ICDD: 00-002-0896) for all the samples except for 100% MoO_3_. The XRD pattern of this cubic Mn_2_O_3_ crystalline phase was characterized by an intense diffraction peak at 32.9°. As shown in Figure 2b, the addition of small amounts of Mo (0.6% MoO_3_) resulted in a very slight shifting of this peak to higher diffraction angles. However, no gradual angle shifting was observed as the amount of Mo increased above 0.6% MoO_3_. Given the very similar ionic radii of Mn^3+^ and Mo^6+^ ions (72 vs. 73 pm), no significant changes in the lattice parameter of Mn_2_O_3_ are expected upon Mo doping, thereby resulting in negligible diffraction angle shifts. At higher Mo loadings (8.7–31% MoO_3_), a fraction of the Mo added ended up forming a new monoclinic MnMoO_4_ crystal phase (ICDD: 00-050-1287) with the main diffraction peak at 25.7°. The intensity of this peak increased with the Mo loading. The 100% MoO_3_ sample showed a very different XRD pattern, characterized by a main peak at 27.4°. This pattern was ascribed to a MoO_3_ crystalline phase (ICDD: 00-001-0706). The diffraction peaks characteristics of this phase were not found for the rest of the samples, allowing us to rule out the presence of MoO_3_ in these materials. Unlike MoO_3_, the Mn–Mo mixed oxide phases are thermally stable at 750 °C [28]. No characteristic peaks corresponding to Mn_2_Mo_3_O_8_ were revealed by XRD [23].

**Figure 3:**
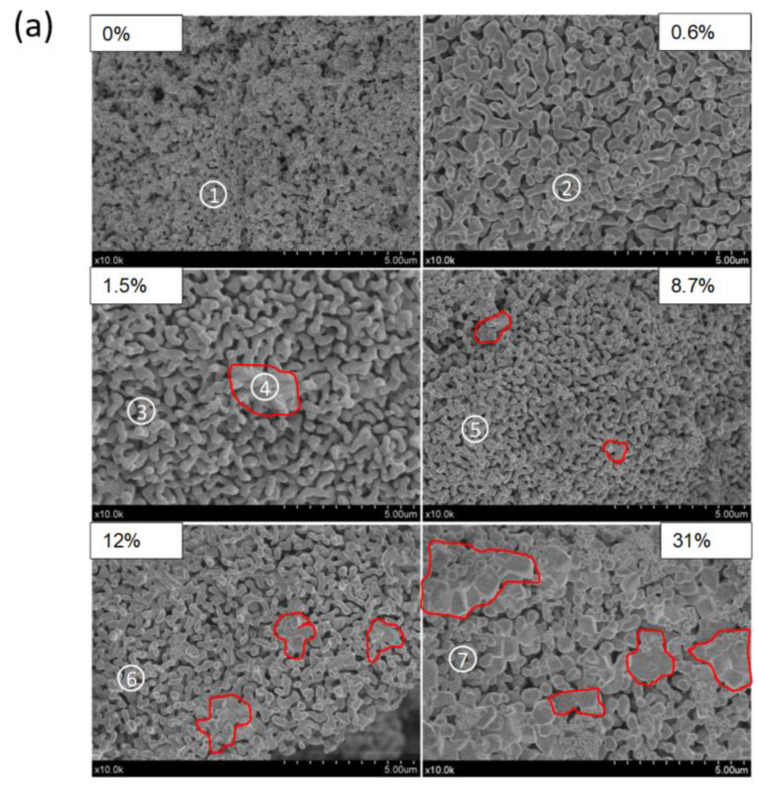

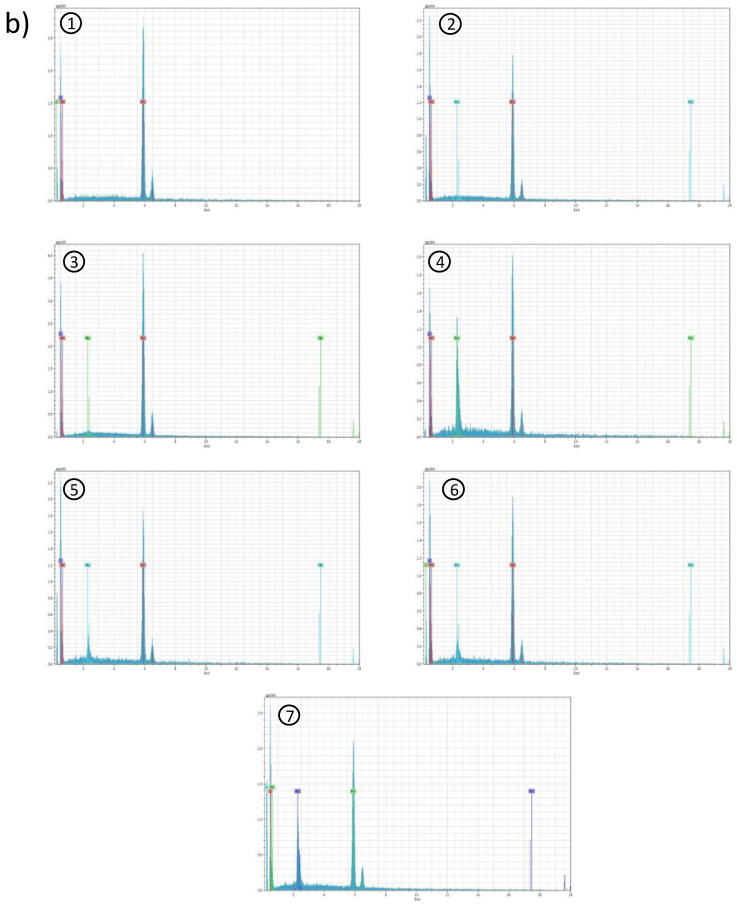
(**a**) SEM pictures of the different Mn–Mo materials studied herein. Some compact secondary particles are encircled in red for clarity. Numbers indicate the spots from which the EDS analyses were obtained. (**b**) EDS spectra corresponding to the spots of Figure 3a.

The morphology of the materials was observed by SEM, and the most representative micrographs are shown in Figure 3a. Remarkably, all the samples except 30% MoO_3_ showed a well-formed coral-like morphology. This coral-like morphology is believed to be key in facilitating oxygen diffusion during the redox process and in preventing excessive sintering of the material at high temperatures. However, as the amount of Mo increased in the samples, the micrographs revealed the presence of compact secondary particles (encircled in red in Figure 3a), which was more evident for the 8.7, 12, and 31% MoO_3_ materials. We carried out energy-dispersive X-ray spectroscopy (EDS) analyses on our samples by performing elemental measurements at different spots of the SEM pictures (indicated by numbers in Figure 3a). The EDS spectra obtained for each of these spots are shown in Figure 3b. These analyses we aimed to confirm the presence of Mo in our samples. These analyses were performed over zones with coral-like morphologies. At these zones, we found Mo contents relatively close to the nominal loadings in all cases except for the 12% MoO_3_ sample, which showed an unexpectedly low value. EDS also allowed us to compare the composition of compact secondary particles with that of the coral-like zones. As shown for the 1.5% MoO_3_ sample (spots 3 and 4 in Figure 3a,b), the compact particle analyzed was enriched in Mo as compared to the coral-like zone, which showed a composition more similar to the nominal loading. This result seems to support the hypothesis that compact particles might correspond to MnMoO_4_, in line with XRD results. However, we note that these compositional analyses based on EDS are not fully representative of the totality of the samples and have a certain variability depending on the spot analyzed.

**Figure 4:**
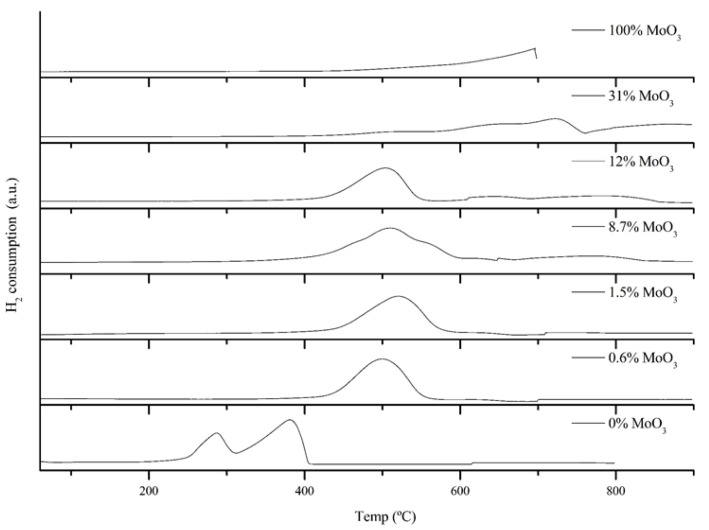
TPR profiles of the Mn–Mo samples under study. Experiments were conducted with 45 mg of sample. T = 60–900 °C (10 °C/min heating rate). Constant 10% H_2_/Ar flow of 50 mL min^−1^.

The TPR profiles of the samples under study are shown in Figure 4. The 0% MoO_3_ sample showed a reduction profile similar to that previously reported for Mn_2_O_3_, with two reduction peaks at 250 and 400 °C [29,30]. Considering the amount of hydrogen consumed, these peaks can be attributed to the successive reduction of Mn^3+^ to Mn(^3+^, ^2+^) and finally to Mn^2+^. Interestingly, the addition of low amounts of Mo (0.6 and 1.5% MoO_3_) changed dramatically the reduction profile of Mn_2_O_3_ from a double-peak to a single reduction peak centered at ca. 500 °C. This dramatic change in the reduction profile suggests that Mo ions could be forming part of the structure of Mn_2_O_3_, strongly affecting the redox behavior of Mn^3+^. The replacement of Mn^3+^ with Mo^6+^ is expected to generate a charge imbalance in the Mn_2_O_3_ lattice, given the different oxidation states of the exchangeable ions. Thus, each Mo^+6^ ion introduced will generate a surplus of positive charges in the lattice. As a result, the Mn_2_O_3_ lattice is expected to generate certain crystalline defects (i.e., one Mn^3+^ vacancy for each Mo^6+^ ion exchanged) to offset the excess of positive charges. These cation vacancies can produce significant changes in the properties of Mn_2_O_3_ even with relatively low doping amounts. Thus, Mn^3+^ cation vacancies generated upon Mo^+6^ doping have been previously reported to result in significant changes in the morphology and redox behavior of manganese oxide materials [27]. As the amount of Mo increases in the samples (8.7–31% MoO_3_), new broad reduction peaks appeared at temperatures higher than 600 °C. These broad reduction peaks could be attributed to the reduction of the mixed phase MnMoO_4_, which was present in these samples according to the XRD patterns. Similar TPR profiles were reported by Cadus et al. for Mn_2_O_3_-supported molybdenum materials [31]. The 100% MoO_3_ sample showed a broad reduction signal between 550 and 700 °C, the temperature at which sublimation was observed and the experiment was interrupted.

### 3.3. Redox Cycling Behavior

**Figure 5:**
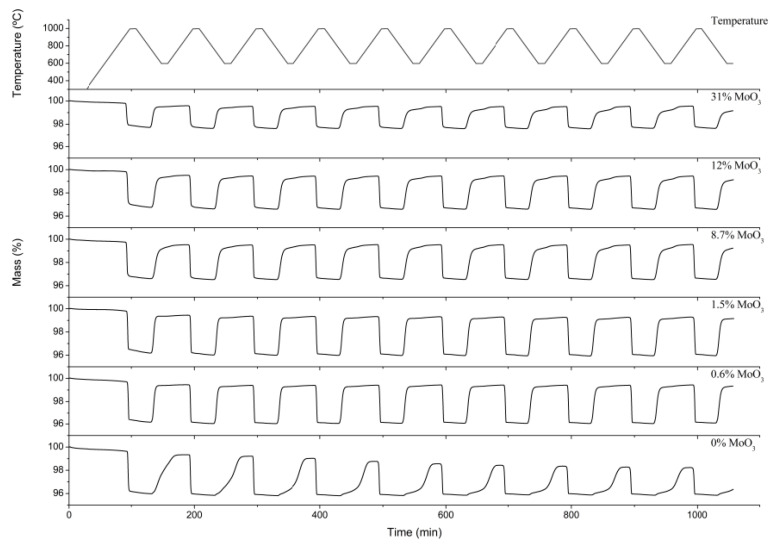
Thermal cycling behavior of the Mn–Mo samples under study for 10 consecutive reduction oxidation cycles.

The redox behavior of the samples was studied by monitoring the losses and gains of weight after consecutive reduction–oxidation cycles, respectively (Figure 5). Thus, a weight loss was indicative of reduction (O_2_ release, reduction, charge), whereas a weight gain was associated to oxidation (O_2_ uptake, oxidation, discharge). As shown in Figure 5, pure Mn_2_O_3_ showed the poorest redox cyclability among the materials studied herein, with very low re-oxidation rates (i.e., slow weight gains). Both the oxidation kinetics and the thermal cyclability improved significantly upon addition of Mo to Mn_2_O_3_. Thus, the Mn–Mo samples showed complete reduction at temperatures below 1000 °C. As shown in Table 2, the weight loss of the samples decreased with the Mo content. We calculated the theoretical weight change considering only the actual amount of manganese in the mixed oxide (determined by ICP-AES, Table 1) and included those values in Table 2. Interestingly, the deviation from the theoretical value increased with the amount of Mo. Thus, the 8.7% MoO_3_, 12% MoO_3_, and 31% MoO_3_ samples showed weight losses above their theoretical value, thereby suggesting that the MnMoO_4_ phase identified by XRD (Figure 2) possessed some redox activity, as suggested by the TPR profiles (Figure 4).

**Figure 6:**
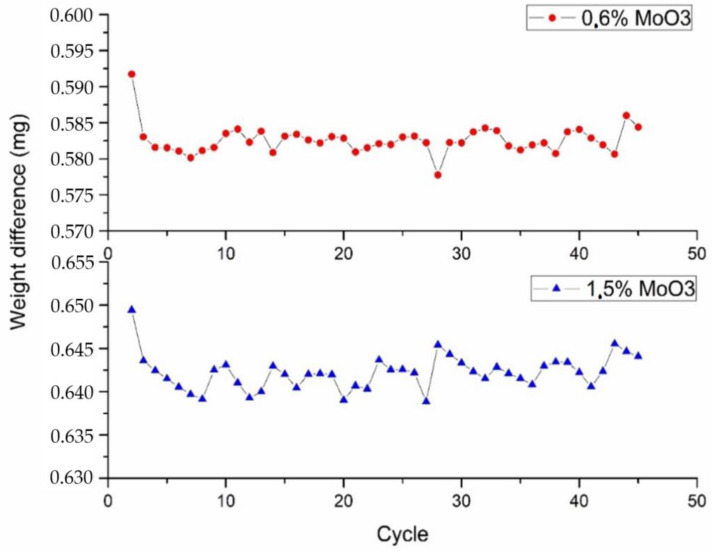
Long-term cyclability tests for the 0.6% MoO_3_ and 1.5% MoO_3_ samples.

The 0.6% MoO_3_ and 1.5% MoO_3_ samples showed the best long-term redox cyclability among the samples studied herein, with no noticeable loss of reactivity after 10 consecutive redox cycles. These samples showed square reduction and oxidation profiles, which are indicative of a stable redox behavior. The addition of Mo above 1.5% MoO_3_ did not enhance the oxidation rate, and a progressive loss of cyclability was observed as the amount of Mo increased in the samples. Thus, additional long-term cyclability tests were performed on the 0.6% MoO_3_ and 1.5% MoO_3_ samples. Figure 6 shows the weight losses of the reduction process for 45 consecutive redox cycles. After the first two cycles, both samples showed excellent cyclability, with no significant changes in the weight losses. The optimum long-term redox behavior of these samples could be ascribed to their coral-like morphology (Figure 3). This morphology allows oxygen diffusion through the solid, thereby facilitating the reduction and oxidation processes. This coral morphology has been previously associated with optimum redox behavior in Mn-containing materials [32]. Interestingly, these samples showed lower surface areas than the rest of oxides (Table 1), which reveals that this parameter does not determine the redox behavior of these materials. The fact that pure Mn_2_O_3_ (0% MoO_3_) also showed a well-formed coral-like morphology and a poor oxidation rates is a strong indicator that Mo necessarily plays a role in improving the oxidation behavior of the 0.6% MoO_3_ and 1.5% MoO_3_ samples. For these samples, the shift of the main diffraction peak of Mn_2_O_3_ to higher angles (Figure 2) suggests that Mo^6+^ ions (73 pm) were able to replace Mn^3+^ ions (72 pm) in the Mn_2_O_3_ lattice, likely occupying six-fold octahedral coordination sites [25,26,27]. The poor oxidation behavior of pure Mn_2_O_3_ has been previously ascribed to the significant structural mismatches existing between the reduced (Mn_3_O_4_, tetragonal or cubic spinels) and the oxidized (Mn_2_O_3_, cubic) phases [8]. Thus, the transition from tetragonal Mn_3_O_4_ to cubic Mn_2_O_3_ involves large structural changes (e.g., lattice shrinking) and atomic rearrangements, which ultimately results in a sluggish oxidation process. The addition of Fe to Mn_2_O_3_ has been reported to improve the oxidation kinetics by promoting the formation of a cubic Mn_3_O_4_ phase, thereby allowing a smoother transition (from cubic to cubic) from the reduced to the oxidized materials [33]. The addition of low amounts of Mo (0.6% MoO_3_ and 1.5% MoO_3_) could produce a similar effect in the reduced Mn_3_O_4_ phase, facilitating the transition from the reduced to the oxidized phases. The TPR profiles of these samples, with a single reduction peak instead of a double-peak profile for pure Mn_2_O_3_ (Figure 4), seem to support this hypothesis. As shown in Figure 5, the addition of Mo in amounts larger than 1.5% MoO_3_ did not improve the oxidation behavior of Mn_3_O_4_, with the oxidation rate decreasing as the amount of Mo increased in the samples. The amount of monoclinic phase MnMoO_4_ in these samples also increased with the Mo loading (Figure 2), which suggests that this phase was not responsible for improving the oxidation behavior of the solids.

Besides the weight variation (which is directly related with the storage capacity), the TES operational temperatures are also very important in determining the efficiency of thermal storage devices for CSP plants [34]. Thus, since reduction and oxidation take place at different temperatures (i.e., thermal hysteresis) for the Mn–Mo samples, heat is stored and released at different temperatures. This thermal hysteresis is one of the main factors affecting to the charge–discharge exergy efficiency, such that it is desirable that these temperatures are as similar as possible, which would result in higher exergy efficiencies [34]:

(4) $${ɳ}_{ex}=\frac{1-\frac{T_{c}}{T_{ox}}}{1-\frac{T_{c}}{T_{red}}}$$

where *T_red_* and *T_ox_* are the temperatures at which reduction and oxidation processes take place, respectively, and *T_c_* is the cold reservoir temperature (298 K). Table 3 summarizes the operational temperatures, the temperature hysteresis, and the charge–discharge exergy efficiency for the 0, 0.6, and 1.5% of MoO_3_ samples (for the redox cycles described in Figure 6). First of all, it is important to remark that the experimental temperatures are slightly higher than the one predicted by the thermodynamic study (Table 1) as result of the critical influence of diffusion and chemical kinetics in the process, which is not considered in the equilibrium calculations [35].

Regarding the operational temperatures, the reduction temperature (i.e., the temperature at which maximum oxygen release rates are reached) increased with the cycle number, while the oxidation temperature (i.e., the temperature at which maximum oxygen uptake rates are reached) decreased upon thermal cycling for both samples, although this was more evident for the 1.5% MoO_3_ sample. This fact implies that the higher the number of cycles the higher the temperature hysteresis, although this parameter seems to stabilize for both materials after the 30th cycle. Additionally, as the change in the operational temperatures is more evident for the 1.5% MoO_3_ sample, it presents a higher decrease in the hysteresis temperature as compared to Mn_2_O_3_. Most importantly, the temperature hysteresis was significantly reduced (by 80–30 and 95–55 °C) for the 0.6% MoO_3_ and 1.5% MoO_3_ samples as compared to Mn_2_O_3_, respectively, thereby enhancing the charge–discharge efficiency of the cycle (by 3.6–2.0 and 4.1–3.0%, respectively).

Additionally, the operational temperatures must be compatible with the working conditions of other parts of the CSP plants such as the solar concentrator (which must concentrate the solar radiation to achieve the required temperatures) and the receiver (where the reactions take place). If we assumed that the solar reactor is a perfectly insulated blackbody receiver, the absorption efficiency is given by Equation (5) [36]:

(5) $$\eta_{\mathrm{abs}}=1-\frac{\sigma \cdot T_{reactor}^{4}}{I\cdot C}$$

where *σ* is the Stefan–Boltzmann constant (5.67·10^−11^ kW/m^2^·K^4^); *T_reactor_* is the solar reactor temperature (*T_red_*); *I* is the direct-normal solar irradiation (taken as 1 kW·m^2^); *C* is the concentration ratio which is defined as the solar radiative flux normalized to 1 kW/m^2^ and is usually expressed in “suns” units. Table 4 summarizes the absorption efficiency for 0.6 and 1.5% of MoO_3_ samples for the different cycles as a function of the concentration ratio of the solar concentrator. Although the temperature hysteresis is different for both materials, the absorption efficiency was almost similar and higher than 90% when the concentration capacity is higher than 2000 suns, which indicates that the operational temperatures of these materials are compatible with current solar technology, and that these materials are promising for large-scale thermochemical heat storage applications.

## 4. Conclusions

The addition of small amounts of Mo to Mn_2_O_3_ was found to improve the TCS behavior (e.g., oxidation kinetics and redox cyclability) of this oxide without significantly increasing its reduction temperature. XRD suggested that most of the Mo added ended up forming a separate monoclinic phase MoMnO_4_, while only a limited fraction of the Mo ions were inserted in the cubic Mn_2_O_3_ structure. Despite the limited extent of this substitution, the presence of Mo^6+^ ions in the structure changed dramatically the redox behavior of Mn_2_O_3_, as revealed by TPR. SEM observations revealed a coral-like morphology, particularly for samples with low Mo loadings. Mo-enriched compact particles and a partial collapse of the coral-like structure were observed for Mo loadings above 8.7% MoO_3_. The 0.6 and 1.5% MoO_3_ samples showed excellent long-term (45 cycles) TCS behavior, with fast oxidation processes and outstanding cyclability. These samples also showed lower temperature hysteresis as compared to Mn_2_O_3_, which resulted in higher exergy charge–discharge efficiencies. The operational temperatures of the Mn–Mo materials prepared herein were compatible with those typical of solar receivers, potentially reaching absorption efficiencies higher than 90% at concentration capacity values typical of CSP plants.

## Figures and Tables

**Table 1:** Theoretical reduction temperature and specific surface area of the samples studied herein.

Sample	Theoretical MoO_3_ Content (wt%)	Actual MoO_3_ Content (wt%)	Theoretical Temperature of Reduction (°C)	BET Surface Area (m²/g)
0% MoO_3_	0	0.00	922	6.17
0.6% MoO_3_	0.6	0.62	927	3.03
1.5% MoO_3_	1.5	1.52	934	2.98
8.7% MoO_3_	8.5	8.70	992	5.65
12% MoO_3_	12	12.2	1019	3.54
31% MoO_3_	30	31.0	1164	2.03
100% MoO_3_	100	100	>1400	-

**Table 2:** Theoretical (based on the Mn content) and TG-measured weight losses for the second reduction cycle for all the samples under study.

Sample	Theoretical Weight Loss (%)	Measured Weight Loss (%)	Deviation (%)
0% MoO_3_	3.38	3.42	4
0.6% MoO_3_	3.33	3.27	−6
1.5% MoO_3_	3.27	3.25	−2
8.7% MoO_3_	2.76	2.84	+8
12% MoO_3_	2.51	2.81	+30
31% MoO_3_	1.18	1.79	+61
100% MoO_3_	-	-	

**Table 3:** Operational temperatures, hysteresis temperature, and charge–discharge exergy efficiency for the 0% (pure Mn_2_O_3_), 0.6%, and 1.5% of MoO_3_ samples.

Sample	Cycle	*T_red_* (°C)	*T_ox_* (°C)	Δ*T* (°C)	ɳ*_ex_* (%)
Mn_2_O_3_	1	950	640	310	89.06
0.6% MoO_3_	2	960	730	230	92.69
	10	980	720	260	91.83
	30	980	700	280	91.02
	45	980	700	280	91.02
1.5% MoO_3_	2	955	740	215	93.20
	10	970	730	240	92.45
	30	975	720	255	91.94
	45	975	720	255	91.94

**Table 4:** Solar receiver absorption efficiencies for the 0.6% and 1.5% MoO_3_ samples.

Sample	Cycle	ɳ_abs_ (%)				
		*C* = 1000	*C* = 2000	*C* = 3000	*C* = 4000	*C* = 5000
0.6% MoO_3_	2	86.90	93.45	95.63	96.72	97.38
	10	86.02	93.01	95.34	96.51	97.20
	30	86.02	93.01	95.34	96.51	97.20
	45	86.02	93.01	95.34	96.51	97.20
1.5% MoO_3_	2	87.11	93.55	95.70	96.78	97.42
	10	86.46	93.23	95.49	96.62	97.29
	30	86.25	93.12	95.42	96.56	97.25
	45	86.25	93.12	95.42	96.56	97.25

## Data Availability

Not applicable.

## Sample Availability

Samples of the mixed oxides are available from the authors.
